# Policy brief Belgian EBCP mirror group Artificial Intelligence in cancer care

**DOI:** 10.1186/s13690-024-01367-5

**Published:** 2024-08-28

**Authors:** Emilie Cauët, Gabrielle Schittecatte, Marc Van Den Bulcke, BE-EBCP mirror group ‘Artificial Intelligence, Valentina Albarani, El Maati Allaoui, Hélène Antoine-Poirel, Sarah Baatout, Glenn Broeckx, Marcela Chavez, Emmanuel Coche, Romaric Croes, Sofie De Broe, Pieter Demetter, Chloë De Witte, Louis Delsupehe, Frederik Deman, Amelie Dendooven, Jennifer Dhont, Gokhan Ertaylan, Thierry Gevaert, Stefan Gijssels, Koen Hasaers, Karin Haustermans, Rudy Hovelinck, Roland Hustinx, Sébastien Jodogne, Lies Lahousse, Steven Lauwers, Stephane Lejeune, Arthur Leloup, Evi Lippens, Benoit Macq, Brigitte Maes, Carlos Meca, Ward Rommel, Roberto Salgado, Bart Schelfhout, Wim Schreurs, Gwen Sys, Lien Van De Voorde, Isabelle Van Den Bulck, Johan Van Lint, Wim Van Roose, Kristel Van Steen, Ad Vandermeulen, Pieter-Jan Volders, Suresh Yogeswaran

**Affiliations:** Department of Epidemiology and Public Health, Sciensano, Cancer Center, Brussels, Belgium

**Keywords:** Artificial Intelligence, Cancer, Imaging Data, Genomics, Data-driven infrastructure, High-Performance Computing

## Abstract

Artificial Intelligence (AI) is already a reality in health systems, bringing benefits to patients, healthcare providers, and other stakeholders in the health care. To further leverage AI in health, Belgium is advised to make policy-level decisions about how to fund, design and undertake actions focussing on data access and inclusion, IT-infrastructure, legal and ethical frameworks, public and professional trust, in addition to education and interpretation. EU initiatives, such as European Health data space (EHDS), the Genomics Data Infrastructure (GDI) and the EU Cancer Imaging Infrastructure (EUCAIM) are building EU data infrastructures. To continue these positive developments, Belgium should continue to invest and support existing European data infrastructures. At the national level, a clear vision and strategy need to be developed and infrastructures need to be harmonized at the European level.

## Introduction

The EBCP (Europe’s Beating Cancer Plan) is a policy-driven initiative running from 2021–2027. It sets out actions to support, coordinate or supplement Member States' efforts at every stage of cancer care. The objective of this policy brief is to outline the major activities and initiatives related to Artificial Intelligence (AI) in health, detail the current activities in Belgium, and conclude with key recommendations to be taken by policy and decision-makers to further implement AI in health.

This policy brief for the federal cabinet of Belgium is being developed to provide a description of the EU cancer initiatives related to AI that Belgium currently participates in, as well as the added value of these projects to the Belgian and EU populations. Each thematic section closes with a discussion on remaining needs and gaps for Belgium in the context of preparing for upcoming the EU4Health and Horizon Europe Work Programmes.

## Methodology

The Belgian EBCP Mirror Group thematic working group (TWG) on AI currently consists of 50 members, with representatives from hospitals, universities, cancer care organisations, patient advocacy and representative organisations, as well as public institutes, and industry representatives. Online group meetings bringing together these different experts were conducted in order to determine priorities and gaps in the AI field related to health.

During these expert discussions, the current status of AI in health, future needs, and priorities in the field of cancer diagnosis and treatment were addressed. The needs and priorities presented in this brief are based on the various experiences of members in the Belgian field, as well as current evidence. The outcomes of the meetings have been distilled by the TWG lead, and members of the Cancer Centre’s, Monitoring and Evaluation Team.

### Issue overview

AI in health is already a reality. Healthcare providers have integrated AI technology into their workflows and decision-making processes. This has already brought improvements for patients, healthcare professionals, healthcare stakeholders, and society at large. Moving forward, generative AI is expected to have a significant impact on patient records standardization and translation across borders. However, the full benefits of AI in health will only be realized if key challenges are addressed, in particular those related to:

- ➢ Data access
- ➢ Data and infrastructure heterogeneity and interoperability
- ➢ Legal matters
- ➢ Ethical aspects
- ➢ Public and professional trust
- ➢ Technology and IT-infrastructure, such as AI tools or High-Performance Computing (HPC)
- ➢ Regulatory and technical matters, such as the definition of common requirements/standards and establishment of best practices, cybersecurity
- ➢ Education and interpretation such as definition of AI-specific reporting guidelines (e.g. how to evaluate quality and compare diagnostic accuracy between studies).

In order to strengthen and integrate EU data research infrastructures, the European Commission launched the development of several major data-driven infrastructures such as the European Health data space (EHDS), the Genomics Data Infrastructure (GDI) and the EU Cancer Imaging Infrastructure (EUCAIM) (see below). Establishing these complex infrastructures demands strong collaboration between experts' teams, clinicians, academics, industry, patient and civil organizations and policymakers. Belgium is actively supporting such initiatives, which are much needed to overcome many of the aforementioned barriers that hamper broad adoption of AI in health.

### Projects & Gaps

#### European *Cancer* Images Infrastructure (EUCAIM)

The EUCAIM project’s aim is to establish and/or upgrade a technical infrastructure necessary to connect national cancer image data sources, including sources in Belgium, to the federated European cancer image data infrastructure [1, 2]. This connection will facilitate training and validation of AI algorithms and prediction models of outcomes using the cancer imaging data available in the infrastructure, and ensure their trustworthiness. The EUCAIM initiative originates from an unprecedented body of work and expertise of the “AI for Health Imaging” Network (AI4HI), which consists of institutions involved in large EU-funded projects on big data and AI in cancer imaging (CHAIMELEON, EUCANIMAGE, INCISIVE, ProCancer-I, PRIMAGE; coordinated by HULAFE, UB, MAG, FORTH and HULAFE, respectively) [2].

Setting up the European Cancer Imaging initiative is one of the flagships of EBCP [3].The EUCAIM project will then showcase how medical images can be accessed, used and pooled in Belgium while strengthening ethical questions on trust in, and security of, personal data protection in full compliance with EU values and rules. It will take important steps towards the EHDS and align with EBCP. The Cancer Image Europe Platform is unique because it will foster the transition from research into clinical practice.

#### Genomic Data Infrastructure (GDI)

The GDI project is enabling access to genomic and related phenotypic and clinical data across Europe. It is doing this by establishing a federated, sustainable and secure data access infrastructure.

The GDI project builds on the outputs of the Beyond 1 Million Genomes (B1MG) and the Genome of Europe (GoE) projects and is realizing the ambition of the 1 + Million Genomes (1 + MG) initiative. To foster opportunities on genomics health research in Belgium, the Belgian 1 + MG Mirror Group was established in 2020 and comprises representatives of all universities, Strategic Research Centers, public health institutes, hospitals, policymakers, patient organizations and industry.

Within the context of the GDI European project, a federated national and local secure data environment for genomic data will be established in Belgium (the Belgian ELIXIR hub). Additionally, Belgium will start generating its own national genomics database (cf. Belgian Genome Biobank and Genome of Europe projects). At the EU level, secure cross-border access to genomic and related health data for European citizens will be established. This initiative, which includes federated search and data access procedures for both research and diagnosis purposes, will lead to groundbreaking insights and continued findings into the role genomics can play in healthcare.

## Policy recommendations

### Data infrastructure

Critical infrastructure is defined as assets or systems essential for maintaining vital societal functions, such as health. We recommend Belgium to continue of the investment and support of existing European data infrastructures. The objective must be to keep working on diverse aspects such as data collection, infrastructure development to connect top cancer research institutions, interoperability guidelines, development of open-source building blocks to implement interoperability standards and AI algorithms, AI model evaluation and dissemination. These efforts are designed to support collaborative research, innovation and development of new AI tools for cancer and ensure a seamless transition into clinical practice.

## Data/model governance

In the context of the above-mentioned European projects, federated national and local secure data research environments will be established in Belgium. However, the biggest barrier to data access may be data governance. The challenge is to make the infrastructures for sensitive data both secure, usable and adhering to the legal and ethical framework. The infrastructures need to support all kinds of users doing cutting-edge analyses across life sciences. Governance must also consider the perspectives of patients and citizens in order to ensure understanding, discussion and acceptance and to offer a non-technical perspective on the potential value and use of AI in healthcare [4]. These specific elements of governance are discussed in this supplement’s accompanying brief on patient and citizen engagement*.*

Therefore, Belgian policymakers need to clarify how existing legislative and ethical frameworks can be made more inclusive so that all forms and applications of AI benefit from the same legal and ethical frameworks. Moreover, it must be considered that enabling access of AI at the research level will require different consent mechanisms, data structures, system architecture and security than enabling access to individual genomes to be utilized in clinical/healthcare setting. These different needs will need to be analysed and addressed.

## Clinical decision making

To leverage AI for clinical decision making, effort should not solely focus on data access and the existence and availability of rich databases of genomic, imaging and health-related data. There should also be a focus on developing various clinical decision support tools based on state-of-the art AI and machine learning approaches, that would help generate patient records and visualisation. Efforts need to be made to ascertain how these new technologies need to be integrated into clinical decision-making.

To achieve the above further research needs to be supported and analyses conducted. First, an analysis on the meaningful use of clinical data, focusing on issues of causality and incompleteness of data must be performed. Second, a conceptual analysis on the clinical employment of the developed AI solutions with a particular focus on workflow and acceptance by decision makers. Finally, a learning needs analysis with key stakeholders in Belgium should be performed for which the finding should be translated into a framework outlining the skills and capabilities health and care professionals need to work with in an AI enhanced environment. Political support would be needed to bring the healthcare, cancer, research and other actors together with the actors working in healthcare education policy to accomplish this.

## Availability of data and materials

Not applicable.

## Abbreviations

AI: Artificial Intelligence
AI4HI: AI for Health Imaging
B1MG: Beyond 1 Million Genomes
EBCP: Europe’s Beating Cancer Plan
EHDS: European Health Data Space
EU: Europe
EUCAIM: European Cancer Imaging Infrastructure
GDI: Genomics Data Infrastructure
GoE: Genome of Europe
HPC: High-Performance Computing
TWG: Thematic Working Group
1 + MG: 1 + Million Genomes
